# Clinical heterogeneity of neuro-inflammatory PET profiles in early Alzheimer’s disease

**DOI:** 10.3389/fneur.2023.1189278

**Published:** 2023-07-31

**Authors:** Dominique Gouilly, Anne-Sophie Salabert, Elsa Bertrand, Marie Goubeaud, Hélène Catala, Johanne Germain, Nadéra Ainaoui, Marie Rafiq, Marie Benaiteau, Jasmine Carlier, Leonor Nogueira, Mélanie Planton, Anne Hitzel, Déborah Méligne, Benjamine Sarton, Stein Silva, Béatrice Lemesle, Pierre Payoux, Claire Thalamas, Patrice Péran, Jérémie Pariente

**Affiliations:** ^1^Toulouse Neuroimaging Center, UMR 1214, Inserm/UPS, Toulouse, France; ^2^Department of Nuclear Medicine, Toulouse Purpan University Hospital Center, Toulouse, France; ^3^Center of Clinical Investigation (CIC 1436), Toulouse Purpan University Hospital Center, Toulouse, France; ^4^Department of Cognitive Neurology, Epilepsy and Movement Disorders, Toulouse Purpan University Hospital Center, Toulouse, France; ^5^Laboratory of Cell Biology and Cytology, Toulouse Purpan University Hospital Center, Toulouse, France; ^6^Critical Care Unit, Toulouse Purpan University Hospital Center, Toulouse, France

**Keywords:** Alzheimer, TSPO, neuroinflammation, neuropsychology, PET scan

## Abstract

The relationship between neuroinflammation and cognition remains uncertain in early Alzheimer’s disease (AD). We performed a cross-sectional study to assess how neuroinflammation is related to cognition using TSPO PET imaging and a multi-domain neuropsychological assessment. A standard uptake value ratio (SUVR) analysis was performed to measure [^18^F]-DPA-714 binding using the cerebellar cortex or the whole brain as a (pseudo)reference region. Among 29 patients with early AD, the pattern of neuroinflammation was heterogeneous and exhibited no correlation with cognition at voxel-wise, regional or whole-brain level. The distribution of the SUVR values was independent of sex, APOE phenotype, early and late onset of symptoms and the presence of cerebral amyloid angiopathy. However, we were able to demonstrate a complex dissociation as some patients with similar PET pattern had opposed neuropsychological profiles while other patients with opposite PET profiles had similar neuropsychological presentation. Further studies are needed to explore how this heterogeneity impacts disease progression.

## 1. Introduction

The relationship between neuroinflammation and the clinical features of Alzheimer’s disease (AD) have been the subject of several positron emission tomography (PET) studies of the translocator protein (TSPO) (1). However, most of these studies have remained controversial in terms of the relationship between cognition and neuroinflammation at the early stages of AD. Cross-sectional studies have shown positive (2), negative (3), and no correlation (4, 5) in AD patients with mild cognitive impairment (MCI). Longitudinal studies have shown that high neuroinflammation at the early stages is associated with either a better (2) or worse (6) cognitive prognosis. These discrepant findings suggest not only that the pattern of neuroinflammation is partly independent of the cognitive profile but also that neuroinflammation is not encapsulated in a stereotyped pattern at the early stages of AD. To elucidate this issue, we performed a cross-sectional study using TSPO PET imaging as proxy of neuroinflammation and a comprehensive multi-domain neuropsychological assessment at the early stages of AD.

## 2. Methods

### 2.1. Patients and clinical assessment

Patients were recruited at the Neurology Department Memory Clinic of Toulouse University Hospital (France). The inclusion criteria were age ranging from 50 to 90 years, amnestic or mixed MCI with mini-mental state examination (MMS) >20/30, and cerebrospinal-fluid AD biomarker evidence (7). The exclusion criteria were evidence of significant co-morbidity, and any other condition with a potential to impact cognition and neuroinflammation. In addition, blood samples were drawn to characterize APOE and TSPO genotypes. Only subjects classified with high (HAB) and mixed (MAB) binding affinity for TSPO were included.

This study was ethically approved (French Ethics Committee “Comité de Protection des Personnes Sud-Est 1”; reference number: 2017–78; French Drug Safety and Health Products Agency; reference number: MEDAECNAT-2018-01-0034). All the participants provided informed consent.

### 2.2. Cerebrospinal fluid biomarkers

Cerebrospinal fluid AD biomarker values were collected as previously described (8) and measured using either ELISA (INNOTEST) or the Lumipulse G1200 system (Fujeribo, Ghent, Belgium) in line with the manufacturer’s procedures. For samples quantified by ELISA, abnormal values were defined as amyloid-β 42 (Aβ_42_) <500 pg./mL or Aβ_42/40_ ratio ≤ 0.05, phosphorylated-tau >60 pg./mL, total-tau >450 pg./mL, according to the cutoff values recommended by the manufacturer, internal data and in the literature (8, 9). For samples quantified with Lumipulse, abnormal values were defined as Aβ_42_ ≤ 600 pg./mL, or Aβ_42/40_ ratio ≤ 0.07, phosphorylated-tau >60 pg./mL, total-tau >450 pg./mL, according to the cutoff values recommended by the manufacturer, and internal data.

### 2.3. Neuropsychological assessment

AD patients underwent a battery of comprehensive neuropsychological tests over 2 days within a week of each other (mean = 7 ± 3 days). Cognitive functions were assessed using the MMS; the free and cued selective reminding test (FCSRT); the delayed-to-matching sample 48 (DMS48) test; the Rey-Osterrieth Complex figure (ROCF) test; forward and backward digit span from the Wechsler Adult Intelligence Scale fourth edition (WAIS IV); the frontal assessment battery (FAB); the trail making test A and B (TMT); the Go/No Go test; the phasic alertness test from the Test of Attentional Performance battery; a measurement of reaction time in neutral condition from the phasic alertness test was used as an assessment of processing speed; the codes from the WAIS IV; 2 min phonemic (p) and categorical (animal) verbal fluency test; a denomination test from the French GREMOTS battery; a test of identical figures identification for gnosis from the French PEGV battery; and the Mahieux’ gestural praxis battery. In addition, behavioral assessment included the State–Trait Anxiety Inventory scale (Stai-y) and Beck’s depression inventory. All these tests and the assessment techniques we used are detailed elsewhere (10).

Furthermore, it was shown that accelerated long-term forgetting pre-dated the objective multi-domain memory impairment of patients with temporal lobe epilepsy or autosomal dominant AD (11, 12). Our team has developed “Mareal” as a new test for accelerated long-term forgetting. Mareal is composed of eight mini-events incidentally interleaved during the first session of neuropsychological assessment (Supplementary Figure S1). Each participant was asked to recall the details of these mini-events 7 day later (see Supplementary Table S1 for details on scoring). In addition, we performed a 7-day delayed recall of the FCSRT. For one patient, the second part of the neuropsychological assessment was performed 3 weeks after the first session because of COVID-19 infection was suspected. For this patient, the accelerated long-term forgetting assessment was performed on day 7 by videoconference.

### 2.4. Neuroimaging acquisition and analysis

#### 2.4.1. MRI

For each subject, brain magnetic resonance imaging (MRI) was acquired on a 3 T MRI scanner (Philips Achieva dStream) including 3D-T1-weighted, fluid-attenuated inversion recovery and susceptibility-weighted imaging. White matter hyperintensities were visually assessed by a trained rater (MP) on the 9-point Fazekas’ rating scale (13). In addition, patients were classified as having possible or probable cerebral amyloid angiopathy by the same rater according to the modified Boston criteria (14). Patients with both lobar and infra-tentorial or deep microbleeds were classified as having mixed angiopathy. One patient had an infra-tentorial microbleed and was classified as having ‘absent’ cerebrovascular co-pathology for compliancy. In addition, two patients had severe artifacts and could not be classified.

#### 2.4.2. TSPO PET imaging

##### 2.4.2.1. Acquisition

A brain PET scanner was performed on a hybrid PET/CT tomograph (Siemens Biograph TruePoint 6.0) within a week of the MRI scan (mean = 7 ± 5 days). The PET was acquired continuously over 60 min after intravenous injection of [^18^F]-DPA-714 (mean = 243 MBq ± 47). All corrections (attenuation, radioactive decay, random, scatter-coincidences and a partial-volume correction) were incorporated in an iterative OSEM reconstruction (3 iterations, 21 subsets). Dynamic data were reconstructed into 32 timeframes (6 × 10 s; 8 × 30 s; 5 × 1 min; 5 × 2 min; 8 × 5 min).

##### 2.4.2.2. Analysis

The use of the SUVR method is non-invasive and more comfortable for participants than a method requiring arterial sampling. In addition, application of the SUVR method seems to increase quantification sensitivity compared to full kinetic modeling and has a high test–retest reliability for TSPO PET imaging in AD (15, 16).

Previous SUVR analysis using [^18^F]-DPA-714 was performed on a 60–90 min interval based on the observation that a compartmental equilibrium of the distribution volume is reached 60 min after injection in healthy individuals (17). In further studies performed on AD patients and healthy individuals, the ratio of the cortical SUV of [^18^F]-DPA-714 relative to the cerebellar cortex was constant on the same interval (2, 18). In our study, we performed a 50–60 min analysis because the SUVR variation was <2% on this interval as previously observed (2, 18). The time stability analysis is shown in Supplementary Figure S2.

Furthermore, one limitation of TSPO PET is the absence of a true reference region. TSPO is expressed in all brain regions (19) and the absence of neuroinflammation from the reference region cannot be predicted with certitude. The cerebellar cortex was validated as a (pseudo)reference region using full quantification and another second-generation TSPO tracer ([^11^C]-PBR-28) (15). The cerebellar cortex was also widely used as a (pseudo)reference region in early AD (2, 4, 5, 18, 20–23). However, a significant uptake was already observed in the cerebellum (3) and there is now evidence in neuropathology, structural and functional imaging, that this region is involved in AD pathophysiology (24–26). Although the use of the whole brain (WB) as a reference region may reduce the amplitude of the SUVR values, it has the advantage of decreasing interindividual variability and highlighting regional variations. Therefore, in addition to an initial analysis using the cerebellar cortex as a (pseudo)reference region, we also replicated all analyses using the WB as a (pseudo)reference region.

##### 2.4.2.3. Processing

T1-weighted images were segmented and spatially normalized using the CAT12 toolbox on SPM12 on MATLAB (v2019b) (27). Reconstructed PET images were corrected for motion before calculation of mean standard uptake value (SUV) parametric images on 50–60 min interval. CT scans were co-registered on T1-weighted images. The transformation thereby derived was applied to SUV PET images to co-register them on T1-weighted images. A binary inclusive mask of pooled gray and white matter segment at *p* > 0.5 was applied. The automated anatomical labeling (AAL3) atlas (28) was deformed to each subject’s MRI native space and mean SUV values were then extracted using the PETPVE12 toolbox (29) on bilateral frontal, temporal, cerebellar cortex (CC), whole brain (WB) and a temporal meta-ROI including the bilateral hippocampus, parahippocampal cortex, amygdala and fusiform gyrus. We calculated the SUV ratio (SUVR) using the mean SUV from the CC or the WB as a (pseudo)reference. Furthermore, SUV PET images were also spatially normalized with the deformation-field used for T1-weighted images. Smoothing was performed (6 mm full-width at half-maximum) after voxel-wise intensity normalization using the CC or the WB as a reference.

### 2.5. Regressions analysis

We performed linear regressions using the following neuropsychological measurements: the total score on the MMS; Mareal, 7-day free and total recall scores; FCSRT, 7-day free and total recall scores, 20-min total recall and immediate total recall scores; DMS48, one-hour delayed recall score; ROCF, 5-min delayed recall score; forward and backward digit span; the total score on the FAB battery; the Go/No Go test, median reaction time; scores on categorical (animal) and phonemic (p) verbal fluency test; phasic alertness index from the phasic alertness test; and the mean reaction time in neutral condition from the phasic alertness test as a processing speed assessment.

In light of cognitive impairment, fatigue or technical issues, some patients did not perform one of the neuropsychological tests. This included one patient for the FCSRT immediate and 20-min recall, three for the ROCF, one for the gnosis test, two for the Go/No Go test, nine for the TMT, three for the WAIS IV codes and one for the denomination test. When these tests were used in statistical analyses, the values for these patients were not considered.

In regional analyses, these neuropsychological measurements were correlated to the SUVR of functionally-related regions including the WB (for the MMS), temporal meta-ROI (accelerating long-term forgetting and anterograde episodic memory scores) and temporal (for the ROCF), as well as frontal regions (working memory, executive functioning, attention and processing speed scores). All regressions were adjusted for age and TSPO genotype. For Go/No Go, processing speed and phasic alertness tests, we used the number of incorrect responses as an additional covariate.

Regional analyses were performed on R software (v1.4.), significance was set at *p* < 0.05, with two-tailed and Holm’s correction for multiple testing when appropriate. In voxel-wise analyses, significance was set at p < 0.05, family-wise error corrected, or at *p* < 0.001 uncorrected using a threshold k of 20 minimum-activated voxels.

## 3. Results

We recruited 29 patients with cerebrospinal fluid biomarker evidence of AD, and MCI (mean MMS = 24 ± 3/30; Table 1 and Supplementary Table S2). There were 12 women (41%), 19 APOE4 carriers (65%), and 13 patients (45%) with diagnosis before 65 years. All patients had impaired memory performance.

**Table 1:** Demographics of AD patients.

Demographics	AD patients *n* = 29
Age, mean (SD)	69 (7.3)
Gender, female, n (%)	12 (41)
TSPO genotype (HAB: MAB), n (%)	16 (55): 13 (45)
Education, years, mean (SD)	14 (3)
Familial history of AD, n (%)	14 (48)
Anti-cholinesterase inhibitors, n (%)	14 (48)
Time from diagnosis (months), median [inter-quantile range]	9 [4–22]
Onset of symptoms before 65 years	13 (45)
APOE 4, n (%)	19 Carriers (65)
Cerebrovascular co-pathology, n (%)	16 Absent (55%) 3 Possible CAA (10%) 4 Probable CAA (14%) 5 Mixed angiopathy (17%)
Fazekas’ white matter hyperintensities score (/9), median [inter-quantile range]	5 [4–7]

APOE, apolipoprotein E; CAA, cerebral amyloid angiopathy; HAB, high affinity binder; MAB, mixed affinity binder; MRI, magnetic resonance imaging; TSPO, translocator protein.

**Figure 1:**
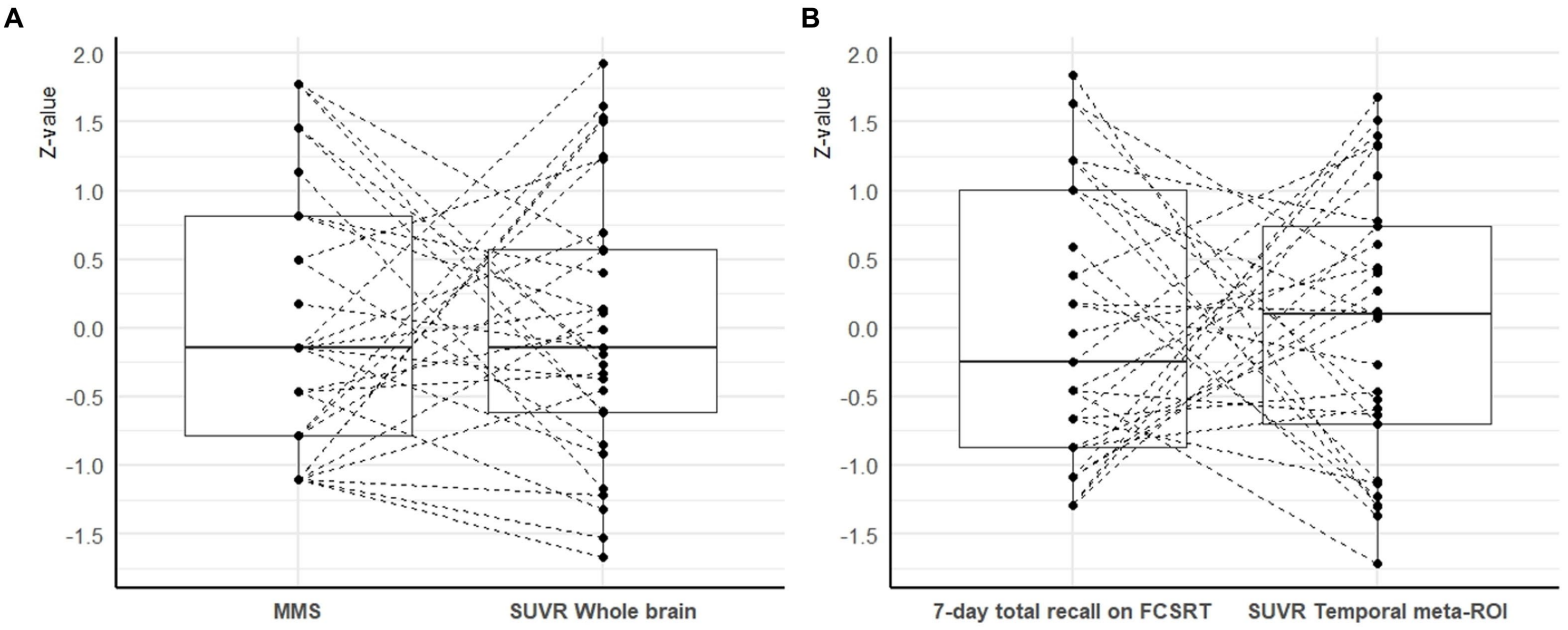
Inter-individual heterogeneity of the relationship between cognitive performances and SUVR values. (Panel **A**) represents the relationship between the MMS score and the whole brain SUVR. (Panel **B**) shows the relationship between the 7-day total recall on the FCSRT and the temporal meta-ROI SUVR. To facilitate comparisons between the distribution of cognitive scores and SUVR values, we calculated z-scores based on the present population (i.e., a null z-value corresponds to the mean of the study cohort). Each line represents one AD patient (MAB or HAB). The reference region was the CC for the whole brain SUVR (panel **A**) and the WB for the temporal meta-ROI (panel **B**). CC: cerebellar cortex; FCSRT: free and cued selective reminding test; MMS: mini-mental state examination; ROI: region of interest; SUVR: standard uptake value ratio; WB: whole brain.

To facilitate comparisons between the distribution of cognitive scores and SUVR values, we calculated z-scores based on the present population (i.e., a null *z*-value corresponds to the mean of the study cohort). We observed that the *z*-values of cognitive scores and SUVR values were distributed along a similar range (Figure 1). However, the relationship between *z*-values of cognitive scores and SUVR values were heterogeneous: patients with cognitive z-values higher than the median had SUVR z-values higher or lower than the median and similarly, for the patients with cognitive z-values lower than the median.

**Figure 2:**
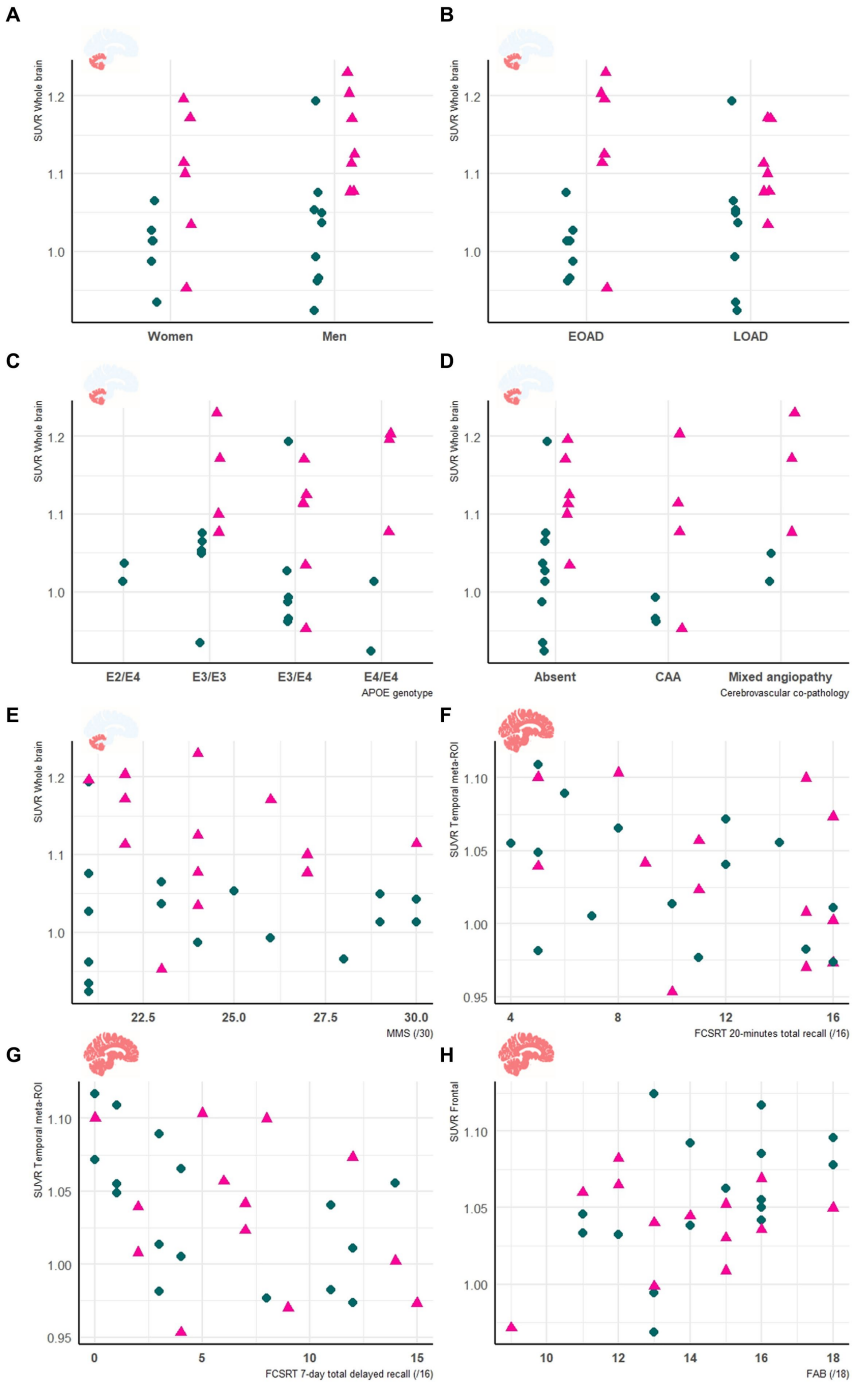
Clinical and neuropsychological features are unrelated to neuroinflammation in early AD. Panels show the relationship between the whole brain SUVR relative to the cerebellar cortex, and sex **(A)**, age at onset **(B)**, APOE genotype **(C)**, cerebrovascular co-pathology **(D)** and MMS score **(E)**. In addition, panels show the relationship between the temporal meta-ROI SUVR using the whole brain as a reference and the FCSRT 20-minutes total recall **(F)** and FCSRT 7-day total recall **(G)**. (Panel **H**) represents the relationship between the SUVR of the frontal area relative to the WB and the FAB score. HAB are represented in green circles and MAB in pink triangles. Age at diagnosis was used as proxy to classify patients as early (<65 years) or late (≥65 years) onset AD (EOAD or LOAD respectively). APOE: apolipoprotein; EOAD: early onset Alzheimer’s disease; FCSRT: free and cued selective reminding test; CAA: cerebral amyloid angiopathy; FAB: frontal assessment battery; HAB: high affinity binder; LOAD: late onset Alzheimer’s disease; MAB: mixed affinity binder; MMS: mini-mental state examination; ROI: region of interest; SUVR: standard uptake value ratio.

We did not find significant corrected correlation between cognitive score and SUVR at voxel-wise, regional, and global level, with neither the WB nor of the CC as reference regions. When removing correction for multiple testing, we observed several correlations in the voxel-wise analysis (Supplementary Table S4). In the regional analysis when using the WB as a reference, we observed three uncorrected negative correlations of the temporal meta-ROI SUVR with the FCSRT immediate total recall (*p* = 0.04; T value = −2.1; β = −0.002; 95%CI = [−0.003 to −0.0001]; adjusted *R*^2^ = 29%); the FCSRT 20-min total recall (*p* < 0.01; *T-*value = −3; *β* = −0.005; 95%CI = [−0.009 to −0.002]; adjusted *R*^2^ = 38%); and the FCSRT 7-day total recall (*p* = 0.01; *T-*value = −2.7; *β* = −0.004; 95%CI = [−0.007 to −0.001]; adjusted *R*^2^ = 38%). In addition, the SUVR values were also not explained by sex (female/male), age at diagnosis (before/after 65 years), APOE phenotype or to the presence of cerebral amyloid angiopathy (Figure 2).

On an individual level, we observed a complex dissociation as the neuroinflammatory PET profiles appeared not to be predictive of patients’ clinical profiles and vice versa (Table 2): some patients with similar clinical presentations had opposed patterns of neuroinflammation, while other patients with opposite clinical presentations showed similar patterns of neuroinflammation.

**Table 2:** Dissociation between clinical and neuroinflammatory PET profiles in early AD.

Clinical findings	CSF and APOE	SWI and T1-weighted MRI scans	Proposition of ongoing neuroinflammatory processes
Case 5: a 64 y.o. man who was referred for a memory complaint. At screening, he had 24/30 MMSE and impairment on episodic memory, denomination and categorical verbal fluency tests. On MRI, multiple lobar microbleeds without hemisiderosis were observed as well as WMH (Fazekas’s score of 8/9) and moderate cortical atrophy.	Aβ_42_: 208 P-tau: 184 T-tau: 1449 APOE E3/E3 TSPO MAB	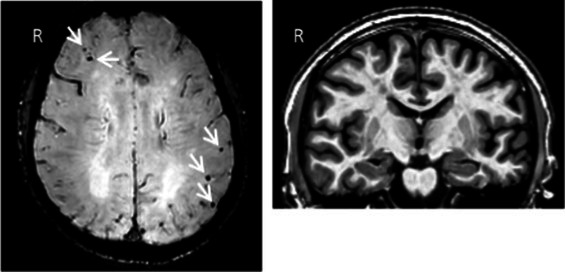	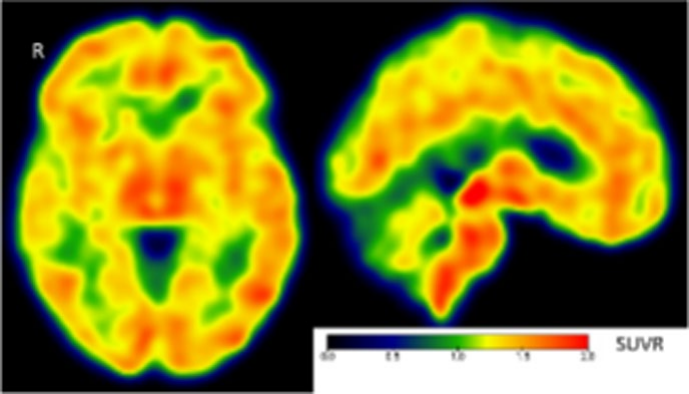 Toxic neuroinflammation associated with mixed angiopathy and AD pathological progression.
Case 21: a 59 y.o. woman with early onset symptoms and familial history of AD. At screening, she had 23/30 MMSE and impairment on episodic memory, executive functions, processing speed and categorical verbal fluency tests. Three lobar microbleeds, WMH (Fazekas’s score of 3/9) and moderate cortical atrophy were observed on MRI.	Aβ_42_: 462 P-tau: 140 T-tau: 768 APOE E3/E4 TSPO MAB	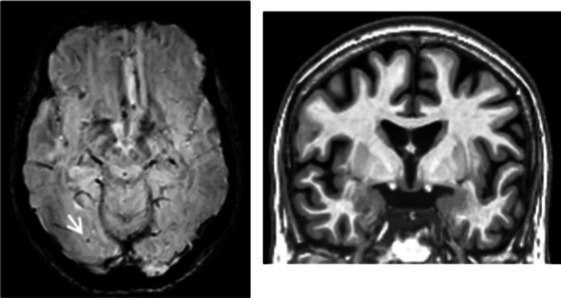	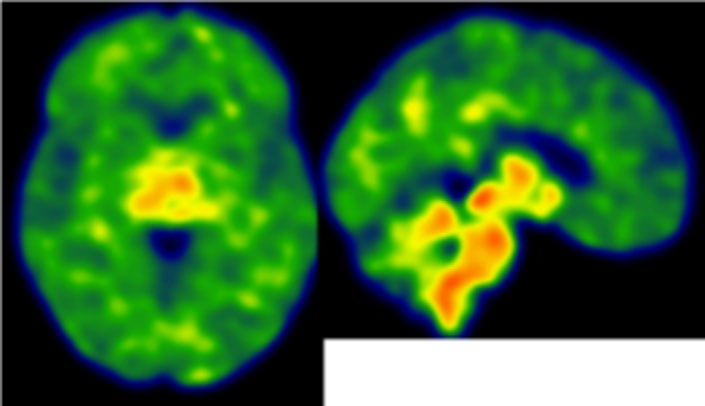 Low cortical neuroinflammation compared to the cerebellar cortex.
Case 2: a 66 y.o. woman with familial history of AD who was referred for a memory complaint. At screening, she had 30/30 MMSE, preserved memory, executive functions and processing speed but encoding impairment in visual recognition memory as well as decreased scores on long-term forgetting tests. Two lobar and one deep microbleed without hemosiderosis, WMH (Fazekas’s score of 5/9) and moderate cortical atrophy were observed on MRI.	Aβ_42_: 327 P-tau: 79 T-tau: 479 APOE E2/E4 TSPO HAB	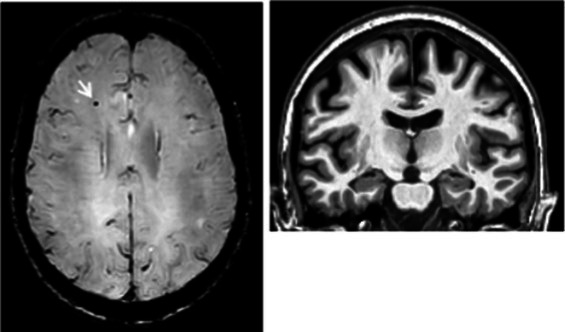	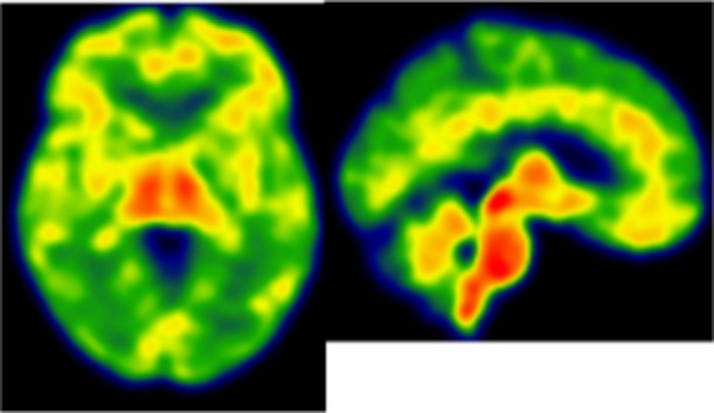 Protective neuroinflammation that might be compensatory to the amyloid load in the frontal and cingulate regions in the absence of spread tau pathology and neurodegeneration.
Case 12: a 64 y.o. man with early onset atypical AD in a posterior cortical atrophy variant. He presented a familial history of AD. At screening, he had 21/30 MMS, multi-domain cognitive impairment, especially constructive apraxia and visual apperceptive agnosia. WMH (Fazekas’s score of 5/9) and cortical atrophy were observed on MRI.	Aβ_42_: 481 P-tau: 103 T-tau: 669 APOE E3/E3 TSPO HAB	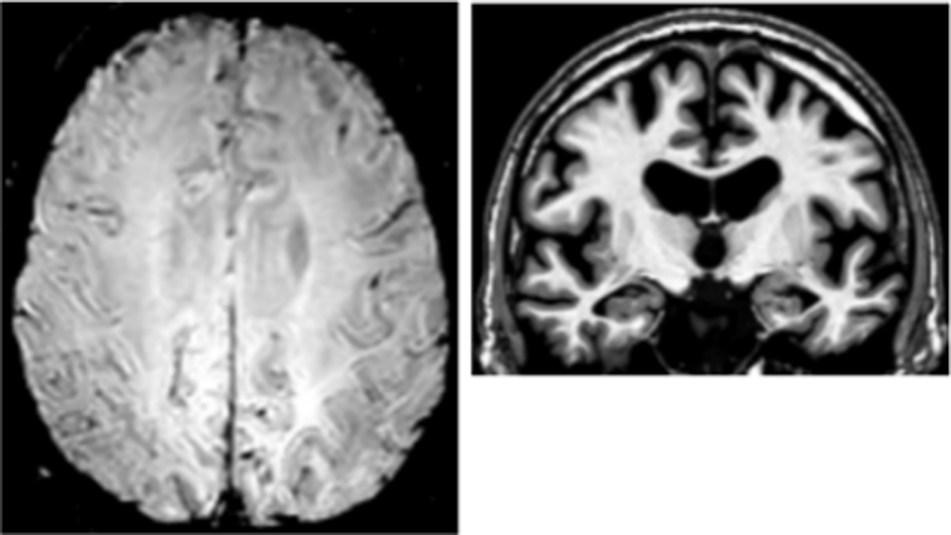	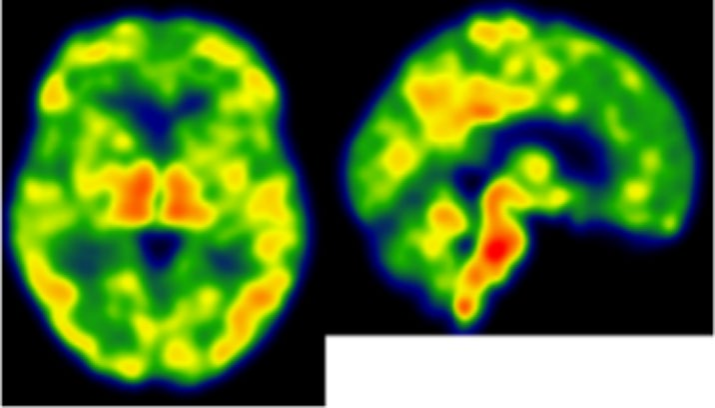 Toxic neuroinflammation associated with AD pathological progression, especially in posterior cortical regions.

All fourth patients are right-handed. TSPO PET imaging showed SUVR relative to the cerebellar cortex and is represented in standard space in the same slice and intensity scale, whereas MRI scans are shown in native space. Cerebrospinal fluid AD biomarker values were abnormal for the four patients (see the method section for details).

Aβ_42_, amyloid-β 42; AD, Alzheimer’s disease; APOE, apolipoprotein E; CAA, cerebral amyloid angiopathy; CSF, cerebrospinal fluid; MMS, mini-mental state examination; MRI, magnetic resonance imaging, P-tau, phosphorylated tau; SWI, susceptibility-weighted imaging; T-tau, total-tau; TSPO, translocator protein; WB, whole brain; WMH, white matter hyperintensities.

## 4. Discussion

This study showed that the pattern of neuroinflammation—as visualized by TSPO PET—is not related to the clinical and neuropsychological profile in early AD. In addition, we found a complex dissociation between the pattern of neuroinflammation and the clinical and cognitive profile: some patients had similar inflammatory PET but opposite neuropsychological profiles, while patients with the same neuropsychological profiles had an opposing intensity of neuroinflammation on PET (Table 2).

These findings may have several lines of explanations. One possibility could be that neuroinflammation exhibits distinct activity independently of the cognitive profile: neurotoxic and driving cognitive impairment for some patients but protective for others reflecting the ability to cope with AD pathology. This might preclude the observation of a linear relationship between neuroinflammation and cognitive performances. However, these opposite relationships with cognitive functioning were already described in a longitudinal study (18) in which patients whose neuroinflammation was high at baseline and stable at follow-up had a better cognitive prognosis compared to those with low neuroinflammation at baseline that increased during follow-up. However, these distinct pathophysiological dynamics are not discernable cross-sectionally on TSPO PET.

Most previously published cross-sectional studies on AD have described negative relationships between TSPO level and the MMS score (3). In our study, we explored the correlation not only with the MMS score but also in relation to multi-domain neuropsychological tests including accelerated long-term forgetting. Therefore, the absence of correlation cannot be due to the mono-dimensionality of the assessment but rather the inter-individual clinical profile heterogeneity. For example, some amnestic patients had agnosia which may have interfered with retention of visual material on the memory assessment and also correlation to neuroinflammation in the temporal region. Another example is that patients with early (<65 years) and late symptoms-onset might have a distinct pathological pattern (30) even though the SUVR values were comparable between these patients (Figure 2). Further studies focusing on the early stages will be needed to explore how distinct clinico-pathological profiles could be associated with neuroinflammation (1, 31).

In our study, we showed that the SUVR was not correlated with APOE phenotype and to the presence and type of cerebrovascular co-pathology (Figure 2). Although these observations should be interpreted with caution, they probably indicate that, to some extent, neuroinflammation might rely on other pathological mechanisms in our results. This is consistent with a recent study showing that APOE phenotype might not influence TSPO level on PET in cognitively unimpaired subjects (32).

Although the participants in our study had evidence of AD cerebrospinal fluid biomarker abnormalities, the use of two distinct quantification methods has precluded their use for statistical analyses. However, AD patients were selected based on early mild amnestic impairment and we observed a tendency toward negative correlations between episodic memory scores and the temporal meta-ROI SUVR. Therefore, our results suggest that neuroinflammation might drive episodic memory dysfunction in early AD although a complex dissociation remains when considering the global clinical and cognitive profile.

The main limitation of this study is the absence of age-matched healthy individuals. However, the presence of neuroinflammation is well-documented and replicated in early AD, especially in the temporal regions (2, 3, 18). In addition, age was added as a covariate in the correlation analyses to correct for aging-related confounds. Besides, we performed the correlation analysis in our study at the voxel, regional and global scale and using two reference regions. No ideal reference region exists for TSPO PET mainly because of the ubiquitous expression of TSPO and the unpredictable nature of the pattern of neuroinflammation (19). The use of the CC as a reference may have biased the analysis if neuroinflammation occurred in this region (Table 2). However, this region was broadly used as a reference region in AD as this area is devoid of mature AD neuropathological changes in the early stages. We also used the WB which may have reduced the inter-individual variability (16) in addition highlighting focal changes. Therefore, it seems unlikely that the low level of neuroinflammation or the choice of reference region can explain the observed results of from our study.

In conclusion, we found evidence of clinical and cognitive heterogeneity of the neuro-inflammatory PET profiles in early AD. Further studies are needed to understand how this heterogeneity is related to disease progression.

## Data availability statement

The raw data supporting the conclusions of this article will be made available by the authors, without undue reservation.

## Ethics statement

The studies involving human participants were reviewed and approved by French Ethics Committee “Comité de Protection des Personnes Sud-Est 1,” reference number: 2017–78; French Drug Safety and Health Products Agency, reference number: MEDAECNAT-2018-01-0034. The patients/participants provided their written informed consent to participate in this study.

## Author contributions

DM, BL, PPa, PPé, CT, and JP performed the conception and design of the study. DG, A-SS, EB, MG, HC, JG, NA, MR, MB, JC, AH, BS, SS, BL, LN, MP, PPa, CT, PPé, and JP performed the acquisition and analysis of data. DG and JP performed the drafting a significant portion of the manuscript and figures. PPé and JP performed the supervision of data analyses, interpretation, and study coordination. All authors participated to the revision of the manuscript and approved the final content of the article.

## Funding

This study was funded by the Fondation Alzheimer (Paris, France).

## Conflict of interest

The authors declare that the research was conducted in the absence of any commercial or financial relationships that could be construed as a potential conflict of interest.

## Publisher’s note

All claims expressed in this article are solely those of the authors and do not necessarily represent those of their affiliated organizations, or those of the publisher, the editors and the reviewers. Any product that may be evaluated in this article, or claim that may be made by its manufacturer, is not guaranteed or endorsed by the publisher.

## Supplementary material

The Supplementary material for this article can be found online at: https://www.frontiersin.org/articles/10.3389/fneur.2023.1189278/full#supplementary-material

## Abbreviations

AD: Alzheimer’s disease
CC: cerebellar cortex
MCI: mild cognitive impairment
MMS: mini-mental state examination
MRI: magnetic resonance imaging
PET: positron emission tomography
SUV: standard uptake value
TSPO: translocator protein
WB: whole brain
