# Supplementary material for: Clinical heterogeneity of neuro-inflammatory PET profiles in early Alzheimer’s disease
Source: Front Neurol. 2023 Jul 31;14:1189278. doi: 10.3389/fneur.2023.1189278 (PMC10425281; doi:10.3389/fneur.2023.1189278)
Supplement: Supplementary file 1 [file Data_Sheet_1.PDF]

Paradigm of Mareal assessment		
First session		Second session
Mini-event 1	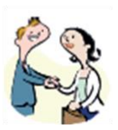	Questioning: What were the conditions of the previous meeting?<br>Expected response: Date, hour, transportation, place, duration.
Mini-event 2	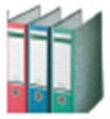	Questioning: Do you remember me asking you to give me any particular object during the meeting?<br>Expected response: The examiner asked the participant to give him/her a green binder<br>lying on a chair behind him/her.
Mini-event 3	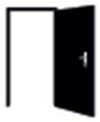	Questioning: Was I absent for a few minutes?<br>Expected response: The examinator was absent for 5 minutes to give a report to another patient.
Mini-event 4	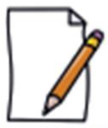	Questioning: Could you describe the test you performed during my absence?<br>Expected response: Puzzle from the WAIS IV.
Mini-event 5	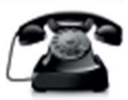	Questioning: Did the telephone ring during my absence?<br>Expected response: The telephone rang two times (4 rings each time, at one-minute intervals).
Mini-event 6	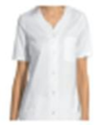	Questioning: Was there something different about me when I returned to the meeting room?<br>Expected response: The gown was changed during the absence.
Mini-event 7	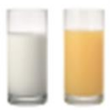	Questioning: Did I offer you something to drink during the meeting?<br>Expected response: A glass of water or orange juice was offered.
Mini-event 8	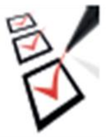	Questioning: Could you describe the test you performed during the entire meeting?<br>Expected response: MMS, FCSRT, DMS48, ROCF, digit span, verbal fluency, praxis and gnosis.

Supplementary Figure 1: Paradigm of the Mareal Test.

Mareal is a new test designed to assess the 7-day retention of mini-events. The eight mini-events shown in this Figure are interleaved with standard tests during the first session of neuropsychological assessment. The second session was performed after one week. The participant waits in the same waiting room as before the first session. No spatial or temporal clue is given and none of the objects used during the first session remain in order to avoid influencing recall. The examiner asks the participant to recall every detail that he/she can during the first session. This free recall phase is subsequently completed by cued recall and then by recognition phase if the participant failed to retrieve part or all of an event. The total recall score was calculated by summing up the free and cued scores. Please note that only the free recall and total recall scores of the Mareal Test were used in this study.

Abbreviations: DMS48: delayed matching-to-sample 48; FCSRT: free and cued selective reminding test; MMS: Mini-mental state examination; ROCF: Rey-Osterrieth Complex figure; WAIS IV: Wechsler Adult Intelligence Scale fourth edition.
