# Supplementary material for: Clinical heterogeneity of neuro-inflammatory PET profiles in early Alzheimer’s disease
Source: Front Neurol. 2023 Jul 31;14:1189278. doi: 10.3389/fneur.2023.1189278 (PMC10425281; doi:10.3389/fneur.2023.1189278)
Supplement: Supplementary file 2 [file Data_Sheet_2.PDF]

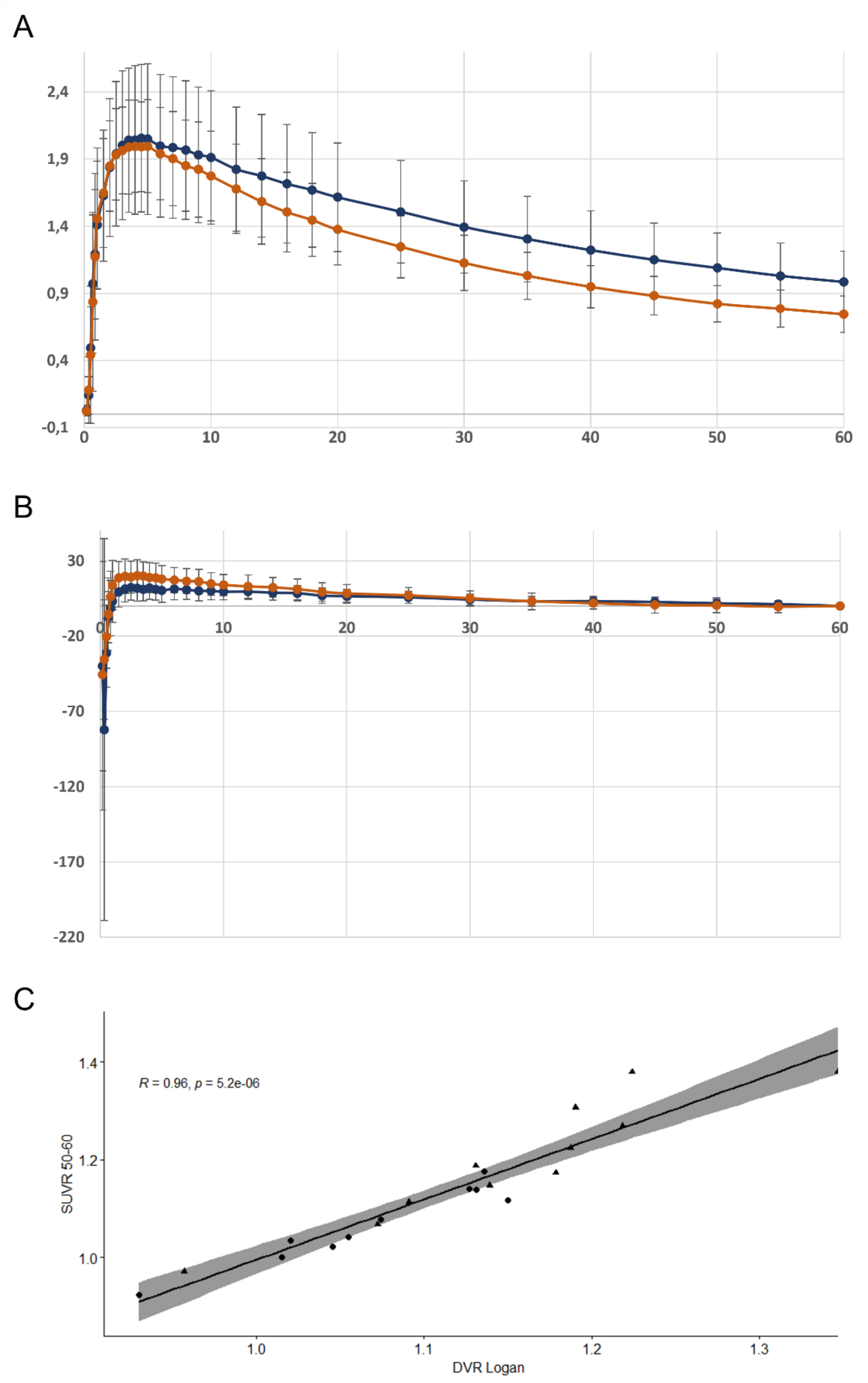

Supplementary Figure 2: Kinetic study of [18F]-DPA714 uptake.

Panel A shows the time-activity curves in the precuneus of AD patients. Values are expressed as the mean ± standard deviation of SUV values (g/mL) for HAB patients in blue (n=16) and MAB in orange (n=13). Panel B shows the variations in SUVR in the precuneus. Values are expressed as the mean ± standard deviation percentage of variation in the SUVR at 60 minutes for HAB patients in blue and MAB patients in orange. The variability between 50 and 60 minutes is inferior to 2.0%. Panel C shows the Spearman's correlation between the SUVR and the distribution volume ratio calculated using the Logan graphical model (HAB in circles, MAB in triangles). As input parameters for the Logan model, we used the k2' estimate from the simple reference tissue model and time t* based on a maximum error of 10%. In this analysis, we only used the individuals for whom standard error of the distribution volume ratio was inferior to 5 (n=21; HAB and MAB together). These analyses were performed on PMOD software (v3.9.) using the PNEURO and PKIN tool, and R software (v1.4.).

Abbreviations: AD: Alzheimer's disease; HAB: high affinity binder; MAB: mixed affinity binder; ROI: region of interest; SUVR: standard uptake value ratio.
