# Supplementary material for: Clinical heterogeneity of neuro-inflammatory PET profiles in early Alzheimer’s disease
Source: Front Neurol. 2023 Jul 31;14:1189278. doi: 10.3389/fneur.2023.1189278 (PMC10425281; doi:10.3389/fneur.2023.1189278)
Supplement: Supplementary file 3 [file Data_Sheet_3.PDF]

Supplementary Table 1: Example of scoring for a mini-event in the Mareal test

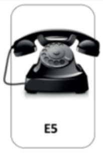	Scoring event 5: Telephone						
	Free recall		Score	Cued recall: *Something special happened while I was away.*	Score	Recognition	Score
	What?	The phone rang		What happened while I was away?		Did the janitor come into the office? Or did the phone ring?	
		Twice		How many times did the phone ring?		Did the phone ring once or twice?	
	Where?	Location of the phone		Where was the phone located?		Was the phone on the desk or on the shelf?	
	Free recall score: … /3			Free & cued recall score: …/3		Free & cued recall & recognition score: …/3	

Example of event 5: During his/her absence, the neuropsychologist makes two phone calls in the examination room (4 rings each time, at 1-minute intervals). The neuropsychologist should count 1 point for each right answer. Please note that the “Recognition score” was not considered in the results presented in this study.
