# Supplementary material for: Clinical heterogeneity of neuro-inflammatory PET profiles in early Alzheimer’s disease
Source: Front Neurol. 2023 Jul 31;14:1189278. doi: 10.3389/fneur.2023.1189278 (PMC10425281; doi:10.3389/fneur.2023.1189278)
Supplement: Supplementary file 4 [file Data_Sheet_4.PDF]

Supplementary table 2: Neuropsychological performances of AD patients.

**Clinical and neuropsychological assessments** mean (SD)	**AD patients** n = 29
MMS score (/30)	24 (3)
**Long-term forgetting**	
Mareal, 7-day free recall (/39)	4 (3)
Mareal, 7-day total recall (/39)	15 (5)
FCSRT, 7-day free recall (/16)	2 (3)
FCSRT, 7-day total recall (/16)	6 (5)
**Anterograde episodic memory**	
FCSRT, 20-minute delayed recall (/16)	11 (4)
FCSRT, immediate total recall (/48)	33 (11)
DMS48, one-hour delayed recall (/48)	42 (6)
ROCF, 5-minutes delayed recall (/36)	7 (5)
**Working memory**	
Forward digit span	6 (1)
Backward digit span	4 (1)
**Executive functioning**	
FAB (/18)	14 (2)
Phonemic (p) verbal fluency, initiation	22 (7)
Categorial (animal) verbal fluency, initiation	18 (8)
TMT B – A, time (s), flexibility	119 (96)
Go/No Go, median reaction time (ms) [mean number of false responses], inhibition	451 (67) [3]
**Attention and processing speed**	
TMT A, time (s)	66 (40)
The codes, WAIS IV (/53)	38 (19)
Processing speed, reaction time (ms) [mean number of false responses]	362 (134) [2]
Phasic alertness, reaction time index [mean number of false responses]	-0.05 (0.1) [8]
**Instrumental**	
Language - Denomination (/36)	31 (7)
Gnosis – Identical figures test (/10)	9 (2)
Praxis - Gestural praxis (/23)	21 (2)
**Behavioral assessment**	
Stai-Y anxiety scale (/80)	44 (10)
Beck's depression inventory (/39)	4 (2)

Values are presented as the mean (SD). Mareal is a new test designed to assess accelerated long-term forgetting (details are shown in Supplementary Figure 1). All the tests presented in this table and the assessment techniques we used are detailed elsewhere (9).

Abbreviations: DMS48: delayed matching-to-sample 48; FAB: frontal assessment battery; FCSRT: free and cued selective reminding test; MMS: Mini-mental state examination; ROCF: Rey-Osterrieth Complex figure; SD: standard-deviation; Stai-y: State-Trait Anxiety Inventory scale; TMT: trail making test: WAIS IV: Wechsler Adult Intelligence Scale fourth edition.
