# Supplementary material for: Clinical heterogeneity of neuro-inflammatory PET profiles in early Alzheimer’s disease
Source: Front Neurol. 2023 Jul 31;14:1189278. doi: 10.3389/fneur.2023.1189278 (PMC10425281; doi:10.3389/fneur.2023.1189278)
Supplement: Supplementary file 5 [file Data_Sheet_5.PDF]

Supplementary Table 3: Statistical parametric mapping of [^{18}F]-DPA-714 SUVR correlation with neuropsychological measurements, family-wise error (FWE) uncorrected.

Neuropsychology	SUVR with the cerebellar cortex as a reference		SUVR with the whole brain as a reference	
	Positive correlation	Negative correlation	Positive correlation	Negative correlation
Global cognitive efficiency				
MMS, (/30)	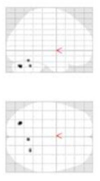	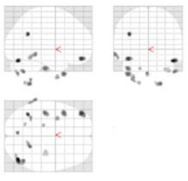	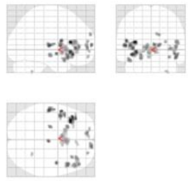	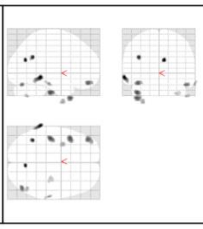
Long-term forgetting				
Mareal, 7-day free recall, (/39)	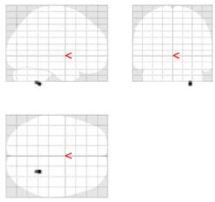	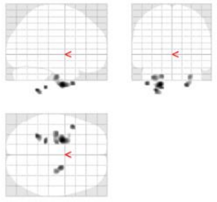	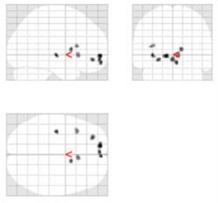	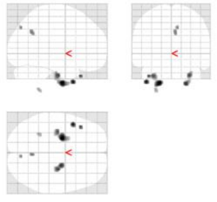
Mareal, 7-day total recall, (/39)	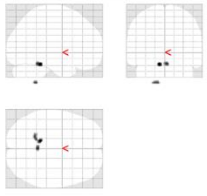	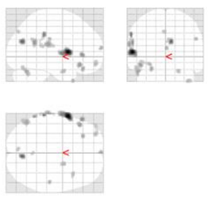	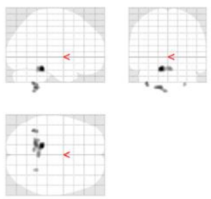	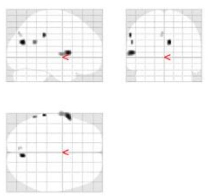
FCSRT, 7-day free recall, (/16)	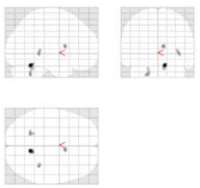		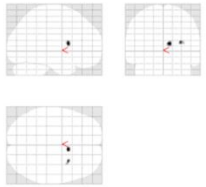	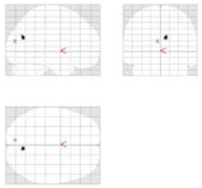
FCSRT, 7-day total recall, (/16)	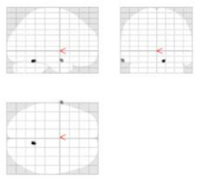	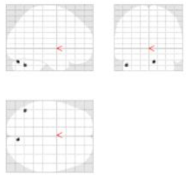	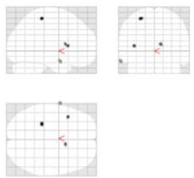	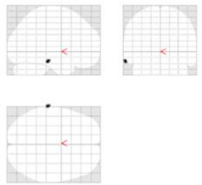

Neuropsychology	SUVR with the cerebellar cortex as a reference		SUVR with the whole brain as a reference	
	Positive correlation	Negative correlation	Positive correlation	Negative correlation
Anterograde episodic memory				
FCSRT, 20-minutes total recall, (/16)	/	/	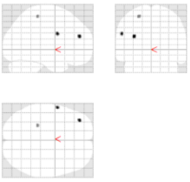	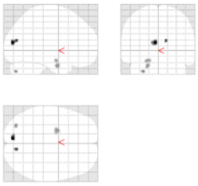
FCSRT, total recall, (/48)	/	/	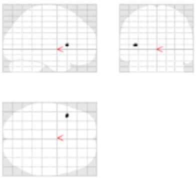	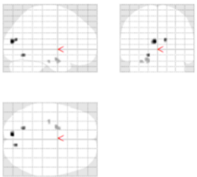
DMS48, delayed recall, (/48)	/	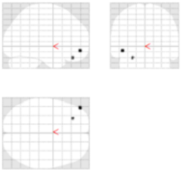	/	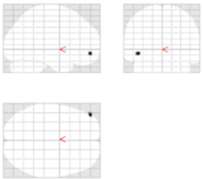
ROCF, delayed recall, (/36)	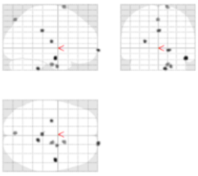	/	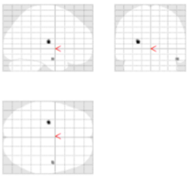	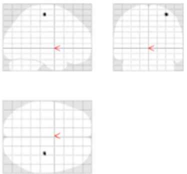

Neuropsychology	SUVR with the cerebellar cortex as a reference		SUVR with the whole brain as a reference	
	Positive correlation	Negative correlation	Positive correlation	Negative correlation
Working memory				
Forward digit span	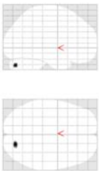	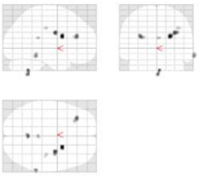	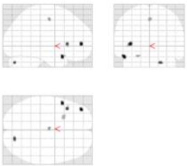	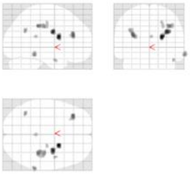
Backward digit span	/	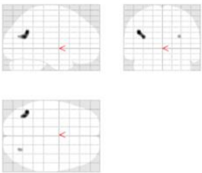	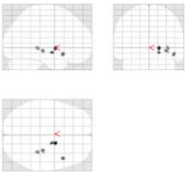	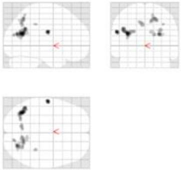
Executive functions				
FAB (/18)	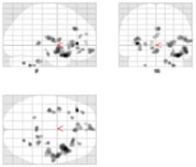	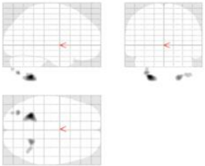	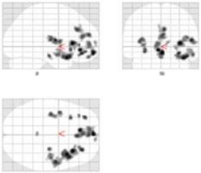	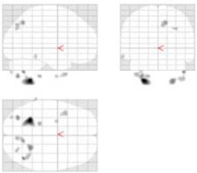
Go/No Go, reaction time (ms)	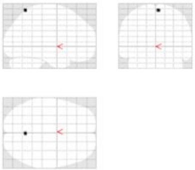	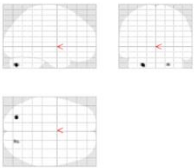	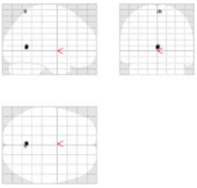	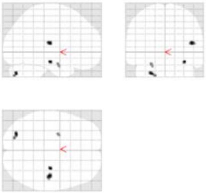

Neuropsychology	SUVR with the cerebellar cortex as a reference		SUVR with the whole brain as a reference	
	Positive correlation	Negative correlation	Positive correlation	Negative correlation
Executive functions				
Categorical (animal) verbal fluency	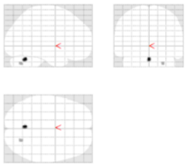	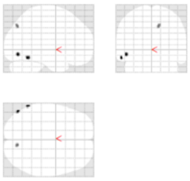	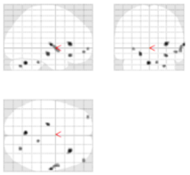	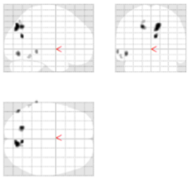
Phonemic (p) verbal fluency	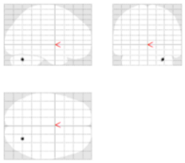	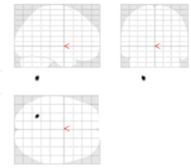	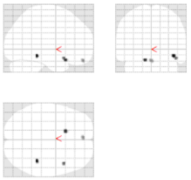	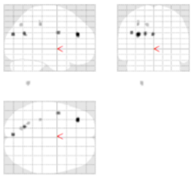
Attention and processing speed				
Processing speed, reaction time (ms)	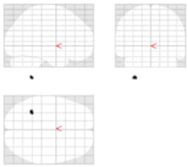	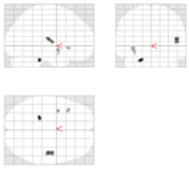	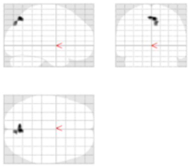	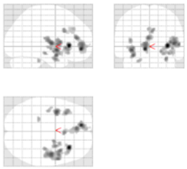
Phasic alterness index	/	/	/	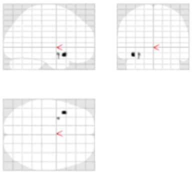

Multiple linear regressions were performed between voxel-wise SUVR and neuropsychological results, FWE uncorrected and adjusted for TSPO genotype and age. For the Go/No Go, processing speed and phasic alertness tests, the number of false responses was used as an additional covariate. The table shows the glass brain images of the correlation results with significance set at p<0.001, FWE uncorrected and k=20. The non-significant results are not shown.

Abbreviations: DMS48: delayed matching-to-sample 48; FAB: frontal assessment battery; FCSRT: free and cued selective reminding test; FWE: family-wise error; MMS: Mini-mental state examination; ROCF: Rey-Osterrieth Complex figure.
